# A Parallel G Quadruplex-Binding Protein Regulates the Boundaries of DNA Elimination Events of *Tetrahymena thermophila*

**DOI:** 10.1371/journal.pgen.1005842

**Published:** 2016-03-07

**Authors:** Christine M. Carle, Hani S. Zaher, Douglas L. Chalker

**Affiliations:** Department of Biology, Washington University in St. Louis, St. Louis, Missouri, United States of America; IMBA, AUSTRIA

## Abstract

Guanine (G)-rich DNA readily forms four-stranded quadruplexes *in vitro*, but evidence for their participation in genome regulation is limited. We have identified a quadruplex-binding protein, Lia3, that controls the boundaries of germline-limited, internal eliminated sequences (IESs) of *Tetrahymena thermophila*. Differentiation of this ciliate’s somatic genome requires excision of thousands of IESs, targeted for removal by small-RNA-directed heterochromatin formation. In cells lacking *LIA3 (ΔLIA3*), the excision of IESs bounded by specific G-rich polypurine tracts was impaired and imprecise, whereas the removal of IESs without such controlling sequences was unaffected. We found that oligonucleotides containing these polypurine tracts formed parallel G-quadruplex structures that are specifically bound by Lia3. The discovery that Lia3 binds G-quadruplex DNA and controls the accuracy of DNA elimination at loci with specific G-tracts uncovers an unrecognized potential of quadruplex structures to regulate chromosome organization.

## Author Summary

Non-canonical DNA structures, including four-stranded Guanine quadruplexes (G4 DNA), have been observed readily *in vitro*, but their regulatory importance within cells has been particularly challenging to demonstrate conclusively. We have discovered a G4 DNA binding protein, Lia3, that specifically regulates programmed DNA elimination events in *Tetrahymena thermophila*. This ciliate deletes nearly one-third of its germline genome from each developing somatic nucleus. These genomic deletion events must be accurate as the thousands of DNA regions excised are located near genes and/or their promoters, thus aberrant excision may alter gene expression. When we knocked out the gene encoding Lia3, we found that the boundaries of the excised regions were heterogeneous for a subset of loci that are flanked by G-rich (5’-AAAAAGGGGG-3’) boundary controlling sequences. When we tested whether Lia3 bound this sequence, we discovered that the sequence itself formed G4 DNA and that Lia3 bound only when the sequence adopted this conformation. Our findings that Lia3 binds G4 DNA and that deletion of the gene encoding Lia3 perturbs the boundaries of the excised loci which are flanked by this quadruplex-forming DNA provides compelling evidence that this non-canonical DNA structure has a critical role during development of these cells.

## Introduction

Ciliates maintain distinct germline and somatic genomes that are partitioned into different nuclei, called micro- and macronuclei, respectively [1]. At each sexual round of the ciliate life cycle, the somatic genome is destroyed, and new germline and somatic genomes are created from identical copies of a zygotic genome formed after exchange of germline nuclei between conjugating partners. The subsequent differentiation of the somatic genome involves massive genome reorganization, which includes fragmentation of the chromosomes and elimination of a large fraction of the germline-derived sequence. In the ciliate *Tetrahymena thermophila*, more than 6,000 dispersed loci, comprising nearly one-third of the genome, are eliminated [2]. These internal eliminated sequences (IESs) consist of both unique and repetitive sequences that are most likely evolutionarily derived from the movement of transposable elements. DNA elimination serves as an effective genome surveillance mechanism that silences these potentially deleterious sequences by removing them from the transcribed nucleus [reviewed in 3].

The eliminated sequences are targeted for excision by small-RNA-directed heterochromatin formation. The targeting small RNAs (called scan RNAs) are produced during meiosis of the micronucleus and then assembled into effector complexes containing the argonaute/Piwi-related protein, Twi1 [4–6]. This mechanism is the evolutionary equivalent of the piRNA pathway, which employs small RNAs to silence transposons in the germline of multicellular organisms [see 7,8]. In *Tetrahymena*, the scan RNA-Twi1 complexes enter developing macronuclei during post-zygotic development and direct histone H3 lysine (K)9 and K27 tri-methylation (me3) to homologous regions [9,10]. The modified chromatin is recognized first by chromodomain proteins Pdd1 and Pdd3 [11,12] and then by additional proteins [13]. Finally, the domesticated piggyBac transposase, Tpb2, excises the IESs [14].

The widespread distribution of IESs throughout germline chromosomes, together with the high gene density of the somatic genome (the average intergenic region is 1 kbp) [15], necessitates accurate removal of the IESs to prevent loss of important coding or regulatory sequences. Previous work has revealed that cis-acting sequences located in the DNA flanking each IES specify excision boundaries [16–19]. Even so, the functionally equivalent controlling sequences of different characterized IESs share no obvious sequence similarity. The best studied of these cis-acting sequences is a polypurine tract (5’ AAAAAGGGGG 3’ or A_5_G_5_) located 45–50 bp outside each excision boundary of the extensively studied M IES [16]. These sequences on each side of the eliminated region reside in opposite orientation such that the G5 portion is proximal to the IES. This A_5_G_5_ tract is both necessary and sufficient to direct accurate excision [16,20]. However, the actual mechanism by which this critical sequence defines the M IES boundaries is unknown.

Here, we show that deletion of the novel gene *LIA3* abolishes accurate excision of both the M IES and other IESs flanked by A_5_G_5_ tracts. Furthermore, we show that the Lia3 protein binds the M IES A_5_G_5_ boundary determinant when it adopts a non-canonical Guanine quadruplex (G4 DNA) structure. G4 DNA forms when Hoogsten base pairs stabilize interactions between four strands each composed of runs of three or more Gs [21]. G4 DNA may form during DNA replication, transcription, or other circumstances that free DNA strands from the double helix; however, *in vivo* evidence for formation of G4 DNA and its regulatory functions is limited. Studies have indicated that cells need to effectively manage sequences that have the potential to form G4 DNA to ensure genetic and epigenetic stability [22,23]. Furthermore, a G4-DNA-forming sequence was found to be critical for antigenic variation in *Neisseria gonorrhoeae*, illustrating that DNA elements that form non-canonical structures are indeed functional [24].

Early evidence that G4 DNA can form in eukaryotic cells came from studies of the telomeres of the multicopy nanochromosomes of *Stylonychia lemnae* in which telomeric G4 DNA and telomere binding proteins were shown to mediate attachment to the nuclear envelope [25,26]. The abundance of *Stylonychia* telomeres permitted ready detection of G4 DNA with the aid of structure-specific antisera. By using a similar approach, G4 DNA was more recently detected *in vivo* in multiple eukaryotic species including in mammalian cells [27–29]. Identification of proteins that bind and/or unwind G4 DNA has provided further evidence that these structures likely serve functional roles *in vivo* [21,30,31]. The *in vitro* binding and *in vivo* genetic data presented here identify a new role for G quadruplexes, in the control of genome-wide DNA elimination, and demonstrate clearly that such non-canonical DNA structures function in genetic regulation.

## Results

### Loss of *LIA3* leads to reduced survival after conjugation

**Fig 1:**
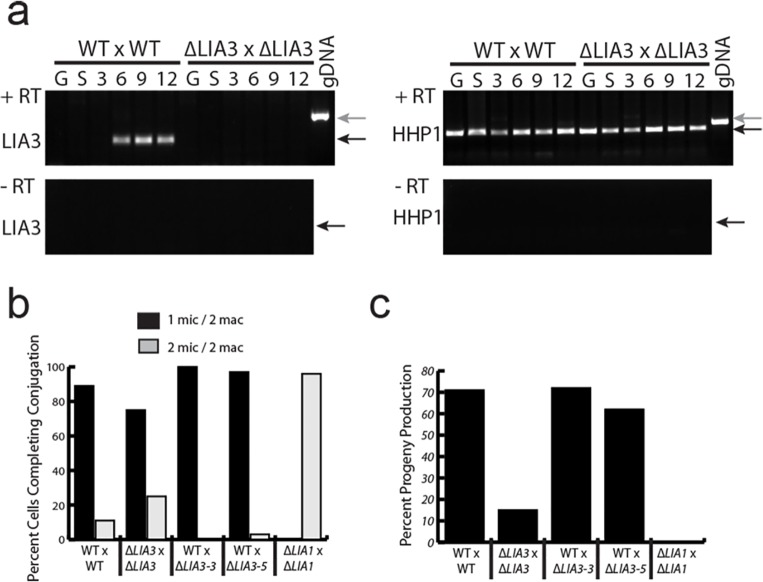
Δ*LIA3* matings have reduced progeny production. (A) rt-pcr of vegetatively growing (G), starved (S), and conjugating cells (3, 6, 9, and 12 hrs postmixing). HhpI is used as a loading control. Black arrows point to cDNA band; grey arrows point to DNA band. Reverse transcriptase was omitted when synthesizing the cDNA for the bottom gels to confirm lack of gDNA contamination in RNA samples. (B) Percent of cells that either complete conjugation by resorbing one of their micronuclei or do not complete conjugation and maintain two micronuclei. (C) Percent of mated pairs that produce viable progeny for each mating. N > 200 for WT x WT and Δ*LIA3* x Δ*LIA3*. N > 45 for WT x Δ*LIA3* matings. For b and c, Δ*LIA1* mating data from [58].

In our search for proteins that are important for the differentiation of the somatic genome, we identified candidates, including Lia3, that are expressed specifically during conjugation and localize to developing macronuclei [13]. Lia3 is a novel protein, which only has obvious similarity with three other *Tetrahymena* proteins of unknown function. To determine whether Lia3 has a critical role in macronuclear development, we created *LIA3* knockout (Δ*LIA3)* strains lacking all germline and somatic copies of *LIA3*. We confirmed the replacement of the *LIA3* coding region with the *neo3* paromomycin-resistance cassette through genetic crosses and Southern blot analysis (S1 Fig), and loss of *LIA3* expression by using rtPCR (Fig 1A). When we mated two *LIA3* knockout lines together, we found that they completed all stages of development, reaching the wild-type (wt) end-point of conjugation, having resorbed one of the two micronuclei (Fig 1B); however, when mated Δ*LIA3* cells were returned to growth media, only 15% of mated pairs produced viable progeny, whereas 70% of wt pairs did so (Fig 1C). These results indicated that *LIA3* participates in, but is not essential for, development.

### *LIA3* is required for correct boundary determination of a subset of IESs

**Fig 2:**
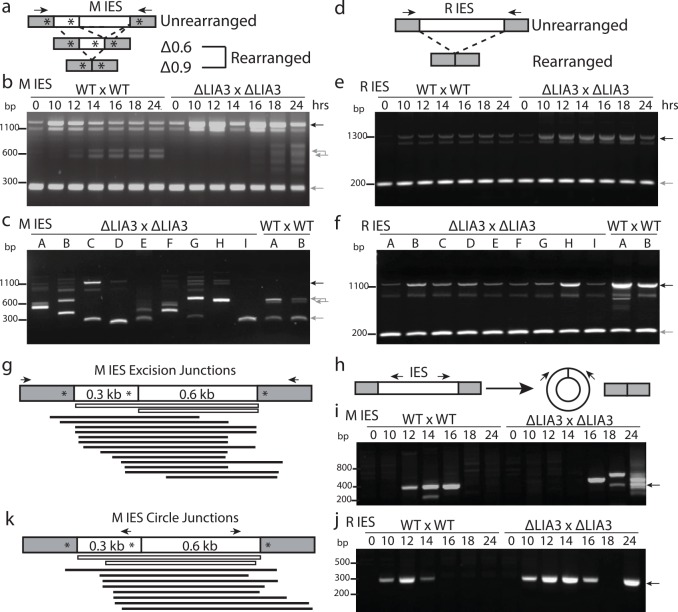
Excision of the M IES is aberrant and delayed in Δ*LIA3* matings. (a and d) Diagram illustrating the alternative rearranged forms of the M IES (a) or R IES d). Grey boxes represent the macronucleus retained flanking regions; white boxes, the 0.6 kb and 0.3 kb eliminated sequences; asterisks, the A5G5 tracts; arrows, the location of the pcr primers used in b and c to amplify across the IES. (b and e) PCR of genomic DNA isolated from different timepoints throughout mating demonstrating that excision of the M IES (b) is both delayed and aberrant in Δ*LIA3* matings while excision of the R IES is normal (e). (c and f) PCR of individual progeny confirming that excision of the M IES (c) is aberrant in Δ*LIA3* matings whereas excision of the R IES (f) is comparable to WT matings. Black arrows point to the unrearranged form and single and double grey arrows point to the expected rearranged forms for b, c, e, and f. In b and c, the doublet observed for the 0.6 kbp M IES deletion reveals that some fraction of wild-type excision events are directed by a cryptic A5G5 tract (AAAGGAGG) rather than the major A5G5 boundary determinant. (g) Diagram displaying the sequenced junctions from individual Δ*LIA3* progeny. A diagram of the M IES denotes the two alterative left boundaries, M1 and M2, and the right boundary M3. Within the IES diagram grey boxes represent the flanking regions; white boxes, the 0.6 kb and 0.3 kb eliminated region of the M IES; asterisks, A_5_G_5_ tracts; arrows, pcr primers. Each thin, white and black boxes beneath the IES diagram represents the region excised from the progeny of either wt or *ΔLIA3* matings, respectively. (h) The strategy to detect excised IES circles: PCR primers located within each IES point outward toward the boundaries and will only amplify a product if the excised region forms a circular intermediate. Grey boxes represent the flanking region; white boxes, the excised region; arrows, pcr primers. (i) PCR results for the excised M IES throughout mating. Primers will not amplify circular products utilizing the interior, right deletion boundary. Note: the lower band in the 14 hr WT mating sample corresponds to an alternative form seen multiple times in WT matings. (j) PCR results for the excised R IES throughout mating. Arrows in i and j point to the expected size of the circular product. (k) Diagram of the excised regions based on sequencing the circular intermediates from Fig 2j.

During macronuclear development, the germ-line derived genome is extensively reorganized and nearly one-third of the DNA is eliminated. To assess whether DNA elimination occurred efficiently in Δ*LIA3* conjugants, we monitored the excision of a well-characterized locus containing two eliminated sequences, the M and R IESs. The M IES exhibits alternative excision, eliminating either 0.6kbp (Δ0.6) or 0.9kbp (Δ0.9) (Fig 2A). By using PCR primers outside the IES, we could detect both rearranged and unrearranged loci (Fig 2B). As all parent lines used in this study possessed only the Δ0.9 form in their macronuclei, detection of the Δ0.6 form during conjugation revealed if and when new excision had occurred in differentiating nuclei. Upon mating wt cells, M IES excision began by 12 hrs of conjugation, evident by a doublet of ~600 bp bands (Fig 2B); In contrast, M IES excision in Δ*LIA3* mating cells was both delayed and aberrant, as newly excised forms were not observed until 16hrs after initiation of mating, and when observed, a ladder of PCR products was visible instead of the doublet (Fig 2B). We did not observe similar aberrancy in R IES elimination due to loss of Lia3. R IES excision may be delayed in Δ*LIA3* matings, as the DNA fragment representing the unrearranged form was more abundant between 10 and 18 hrs than in wt, but this could not be unambiguously determined because *de novo* rearrangement of this IES cannot be distinguished from the DNA present in the parental macronuclei (Fig 2D and 2E). Nevertheless, no aberrant excision was evident, suggesting that the loss of *LIA3* affects the accuracy of excision of only one of these two IESs.

We initially observed aberrant M IES excision in Δ*LIA3* mating populations for which only a portion of cells survived. To determine whether the defective excision detected occurred primarily in the fraction of the population that died, we also examined M and R IES excision in individual surviving progeny cells. The nine individual progeny lines from Δ*LIA3* crosses examined possessed an array of M IES excision products, which reflects the aberrancy observed within the full mating population (Fig 2C and 2B). Excision of the R IES again appeared to be largely unaffected (Fig 2F). Thus, aberrant excision was not limited to the Δ*LIA3* progeny that died as cells with heterogeneous excision boundaries survived conjugation.

To determine how IES boundaries are positioned in the absence of Lia3, we cloned and sequenced a number of the M IES junctions of wild-type and Δ*LIA3* progeny. The boundaries of eliminated DNA can be positioned hundreds of base pairs upstream or downstream of the major wild-type boundaries (Fig 2G). This aberrant elimination could occur due to improper cleavage of the genome by Tpb2 or, alternatively, cleavage could be normal, but the rejoining that must occur subsequent to cleavage could be perturbed. We took advantage of the observation that excised IESs will circularize to map presumed sites of cleavage in both wt and mutant cells [32,33]. The junctions of these circular, excised IESs were recovered by using PCR primers complementary to the excised regions to amplify outward across the joined ends, thus allowing us to map excision boundaries (see Fig 2H). In wt matings, circular products were observed starting either at 10 or 12 hrs into conjugation. In Δ*LIA3* matings, R IES circular products of the predicted size were detected at the same hour into conjugation as they were found in wt matings, but M IES circular products appeared much later and were variable in size (Fig 2I and 2J and S1 Table). The IES boundaries of these circular products showed similar map positions as the boundaries of the rejoined DNA in progeny (Fig 2B and S1 Table). This observation is consistent with aberrancy in the cleavage of the M IES from the genome.

### Lia3 interacts with a polypurine tract found in the flanking region of specific IESs

**Fig 3:**
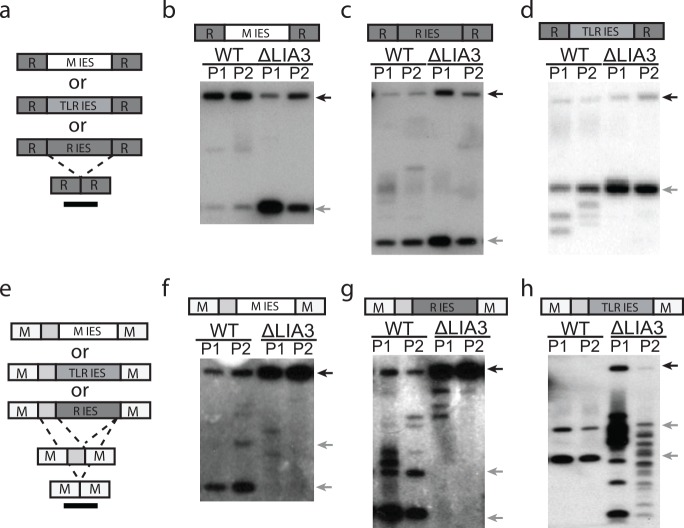
Lia3 acts on the flanking region of the M IES. (a and e) Schematic of the expected rearrangement in wt cells of chimeric IESs used in the Southern blots shown in b, c, e, and f. Boxes labeled M or R represent the flanking DNA of the M IES or R IES; light grey boxes, the 0.3kb internal region of the M IES; box labeleled M IES or R IES or TLR IES, the indicated germline-limited sequence; black bar, Southern probe (b-d, f-h). Each lane of the Southern blot contains genomic DNA isolated from three co-cultured transformants. The diagram above each Southern blot denotes which chimeric IES-rDNA vector was introduced into mating cells. Black arrows indicate the position of the unrearranged DNA, while grey arrows point to fragments of the size expected after accurate IES excision.

To determine why loss of *LIA3* affects M IES but not R IES excision, we tested whether the perturbation in Δ*LIA3* mutants was due to impaired recognition of the M IES or specification of accurate boundaries. Recognition of IESs occurs when complementary scan RNAs match regions of the developing somatic genome and mark them for elimination [4,34], whereas boundaries are determined by sequences flanking each IES, which are retained after excision [16–19]. Two lines of evidence indicate that the M and R IESs differ from one another in both their recognition requirements and boundary determinants: 1) Ema1, an RNA helicase that participates in scan RNA/Twi1 recognition, is required for M but not R elimination [35]; and 2) M and R IESs have functionally distinct and incompatible boundary-controlling sequences [16,17,20]. To determine whether Lia3 acts to identify the eliminated region of the M IES or its flanking boundary sequences, we generated chimeric IESs and tested their excision in both wt and Δ*LIA3* conjugants. These chimeras contained the eliminated region of either the M, R, or a segment of the transposon-like TLR IES [36,37] inserted between either the M or R boundary-controlling flanking sequences. These chimeras were introduced into conjugating cells during nuclear differentiation on rDNA-based replicating vectors, and IES excision was monitored by Southern blot analysis of the transformant DNA. When any of the eliminated sequences, including that of the M IES, was positioned between the R IES’s flanking sequences, the chimera was accurately excised using the normal R IES boundaries, even in Δ*LIA3* matings (Fig 3A–3D). In contrast, when any of these IESs was positioned between the M IES’s flanking DNA, each IES was correctly and efficiently excised in wt matings, but not in Δ*LIA3* matings (Fig 3E–3H). We observed a significant decrease in excision efficiency for the M-flanked M and R IESs (Fig 3F and 3G), whereas the M-flanked TLR IES was efficiently deleted, but its excision lacked clearly defined boundaries, evident as a ladder of products (Fig 3H). The decrease in excision efficiency that we observed in *ΔLIA3* progeny coincides with the decreased M IES excision observed upon deleting or otherwise mutating polypurine tracts flanking the M IES [16,20]. These experiments demonstrate that Lia3 acts in concert with the M IES flanking DNA to specify the boundaries of excision, but does not discriminate between the different IESs placed between these controlling sequences.

**Fig 4:**
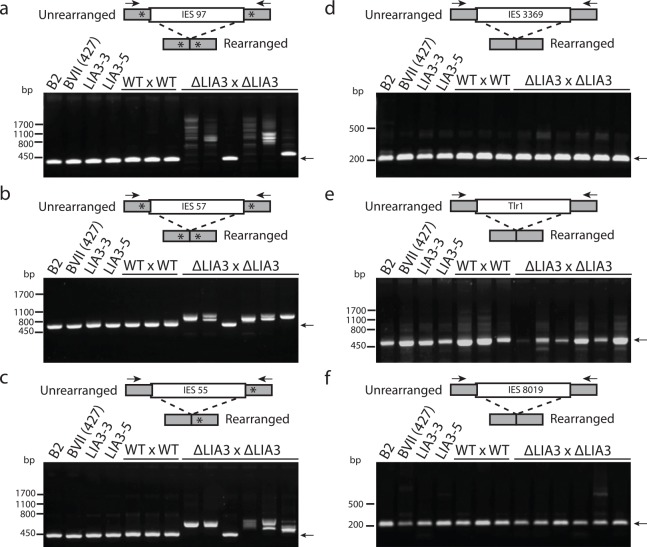
Δ*LIA3* lines aberrantly rearrange IES flanked by A_5_G_5_. Lanes 1–4 are from the wt and ΔLIA3 parent lines used for the matings in lanes 5–13. (a-c) PCR across rearrangement junction of IES flanked by A_5_G_5_ that display aberrant rearrangement in Δ*LIA3* matings. (d-f) PCR across rearrangement junction of IES not flanked by A_5_G_5_ that rearrange normally in Δ*LIA3* matings. Black arrows, pcr primers; asterisks, polypurine tracts; grey boxes, flanking sequences; white boxes, IES. IES 55 has a sequence resembling a polypurine tract only on its right flank.

The boundary-controlling flanking sequences of the M IES consist of polypurine tracts, 5’-A_5_G_5_-3’, located approximately 45bp away from the major boundaries [16]. This A_5_G_5_ sequence is not present in the flanking region of the R IES, leading to our hypothesis that Lia3 interacts with this polypurine tract to determine the position of each excision boundary. If true, Lia3 represents the first protein known to position these boundaries. We first tested this possibility by identifying other IESs with similarly positioned polypurine tracts and assessed whether their excision was aberrant in Δ*LIA3* progeny. All four additional IESs with polypurine tracts located near their boundaries exhibited aberrant excision in progeny of Δ*LIA3* matings (Figs 4A, 4B and 4C and S2A) whereas the several other IESs tested that lacked obvious polypurine tracts were not affected by loss of *LIA3* (Figs 4D, 4E and 4F and S2B–S2F). These findings are consistent with our analysis of chimeric IESs (Fig 3) that showed that diverse IESs are affected by loss of *LIA3* only when flanked by G-rich polypurine tracts. Thus Lia3 appears to specifically control the excision boundaries of a class of IESs containing flanking polypurine tracts.

### Lia3 directly binds to a parallel G-quadruplex formed by a polypurine tract *in vitro*

**Fig 5:**
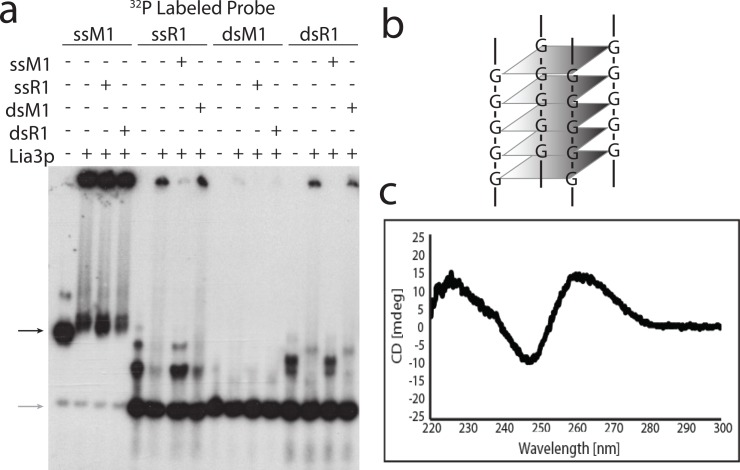
Lia3 binds M IES flanking DNA, which adopts a quadruplex structure. (a) Competition gel shift in which 300 nM His-Lia3 was incubated with 5 times as much unlabeled competitor oligo (listed on the left) followed by incubation with ^32^P-labeled oligo (listed above) before loading on a 4% native gel. (b) Schematic representation of a G4 DNA structure. (c) CD spectrum of the M1 oligo in 100 mM KCl buffer.

The obvious interpretation of our data is that the novel protein Lia3 directly binds to the M IES polypurine tracts and controls the extent of excision. To test the ability of Lia3 to bind DNA, we purified the protein after expression in *E*. *coli* (S3 Fig) and used it in electrophoretic mobility shift assays (EMSA). Initially, we incubated Lia3 with single stranded (ss) or annealed (ds) 30 nt oligonucleotides corresponding to either the leftmost M IES boundary (M1), centered on the A_5_G_5_ tract, or the equivalent region from the leftmost R IES boundary (R1) (Table 1). Lia3 bound strongly to ssM1 and weakly or not at all to the dsM1, ssR1, and dsR1. To confirm specificity, we used unlabeled oligonucleotides to attempt to compete away weaker interactions. The ssM1 oligonucleotide competed effectively for the initial binding observed when using the ssR1 and dsR1 substrates, whereas the ssR1 and dsR1 oligonucleotides could not compete for the binding to ssM1, indicating that the interaction that Lia3 had the highest affinity for the ssM1 probe (Fig 5A).

**Table 1:** 

Oligo Name	Sequence (5’-3’)	G4^1^	Comments
M1	TTTATTAATCAAAAA**GGGGG**TAAATAATAA	Yes	
M1-mut	TTTATTAATCAAAAA**G**C**GGG**TAAATAATAA	No	
M1-C3	TTTATTAATCAAAAACCC**GG**TAAATAATAA	No	Disrupts excision in vivo
M2	TTCAAGACAAAAAAA**GGGGG**ATGGGTTTCC	Yes	
R1	TTTTAAACAGTGTAAAACCCAAAAAGCTAA	No	
Telomere	TT**GGGG**TT**GGGG**TT**GGGG**TT**GGGG**	Yes	*Tetrahymena* telomere
M1-Rv	TTATTATTTACCCCCTTTTTGATTAATAAA	NT	Reverse complement for M1
R1- Rv	TTAGCTTTTTGGGTTTTACACTGTTTAAAA	NT	Reverse complement for R1
M2-Rv	GGAAACCCATCCCCCTTTTTTTGTCTTGAA	NT	Reverse complement for M2

^1^ NT- Not Tested

In these assays, the majority of the unbound ssM1 oligo exhibited an altered electrophoretic mobility, migrating significantly slower than expected, and it was this form of the probe to which Lia3 preferentially bound (Fig 5A, black arrow). This purine-rich oligonucleotide contains a run of Gs, leading us to test the possibility that the probe had adopted G4 DNA structure (Fig 5B). G4 DNA is known to form readily in the presence of KCl, but poorly in the presence of LiCl or without cations [38], so we denatured the oligonucleotide by boiling in either 10 mM Tris-HCl (ph 7.5) alone or supplemented with either 100 mM KCl or 100 mM LiCl, then slow cooled to room temperature before native gel electrophoresis. The M1 oligonucleotide migrated as expected for ssDNA in buffer without salt or with LiCl, but abnormally slow in the presence of KCl (S4A Fig). Mutation of either the first three Gs within the A_5_G_5_ segment to Cs, mutations known to abolish boundary function [20], or even simply changing the second G to C, was sufficient to prevent the M1 oligo from forming a higher-ordered structure (S4 Fig). All these observations are consistent with a G4 DNA structure. We confirmed that 30 nt, A_5_G_5_-containing oligonucleotides representing either the M1 and M2 flanking region (the sequences from the two alternative left side M IES boundaries centered around the A_5_G_5,_ [16]) formed quadruplex structures by performing circular dichroism (CD) [39] (Figs 5C and S4). Parallel G4 DNA exhibits a diagnostic positive peak at 260nm and a negative peak at 240nm [39]. Both the M1 and M2 oligonucleotides displayed CD spectra diagnostic with formation of parallel G4 DNA when in the presence of KCl, but not, at least for M1, when in the presence LiCl (S4 Fig). This observation further supports our conclusion that these probes formed a quadruplex in the conditions used in our EMSA.

**Fig 6:**
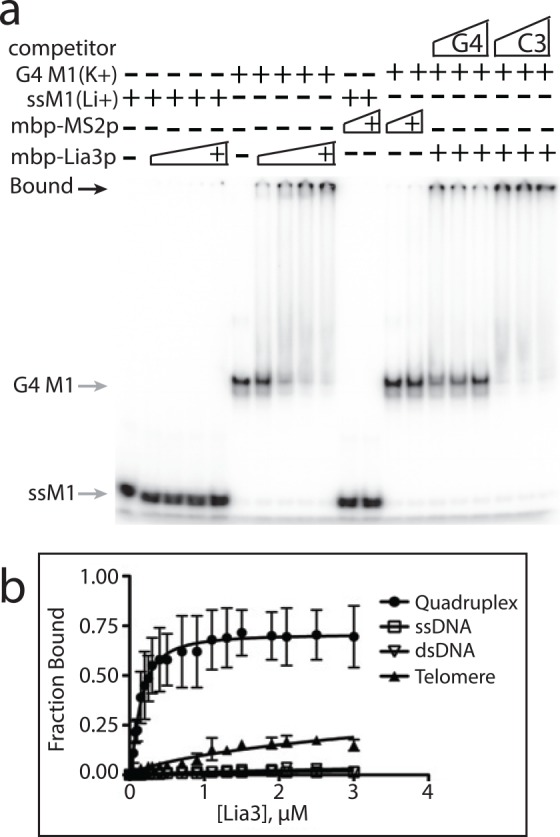
Lia3 binds a parallel G-quadruplex forming oligonucleotide *in vitro*. (a) 50–400 nM MBP-Lia3 was incubated with ssM1 (LiCl) or G4 M1 (KCl) probes and binding was analyzed on 5% polyacrylamide gels. Increasing amounts of either wt M1 or C3G2 mutant M1 oligonucleotide was added to assess competition in the presence of 400 nM MBP-Lia3. (b) Binding curves as determined by EMSA for His-Lia3 binding to ssM1, dsM1, G4-M1, and G4-*Tetrahymena* telomere.

To rule out the possibility that a co-purifying contaminant in the extract was responsible for quadruplex binding, we performed parallel purification of Lia3 and the MS2-coat protein and used each in EMSA. Our initial binding assays were performed with a histidine-tagged Lia3 protein, which required denaturing lysis to recover from *E*. *coli*. We subsequently expressed Lia3 with a maltose binding protein (MBP) fused to its amino terminus, which allows purification in non-denaturing conditions, and isolated MBP-Lia3 along with MBP-MS2 (S5 Fig). The MBP-Lia3 specifically bound the G4 DNA M1 probe formed in the presence of KCl, but not the ssMI probe (in LiCl) (Fig 6). The specificity of Lia3 for G4 DNA is further revealed by the observation that none of the residual ssM1 DNA (lower band) remaining in KCl treated probe samples was shifted upon addition of protein (Figs 5 and 6). The MBP-MS2 protein bound neither the probe in LiCl or KCl. Excess unlabeled M1 oligonucleotide, but not the C3G2 mutant oligonucleotide, could compete away MBP-Lia3 binding, which further supports that Lia3 preferentially binds G4 DNA.

To further assess the specificity of Lia3 for parallel G4 DNA, we measured binding affinity of Lia3 to M1 G4 DNA, ssM1, dsM1, or *Tetrahymena* telomere sequence, which is known to form mixed quadruplex structures (Figs 6B and S6). We determined that the Kd of Lia3 for the M1 quadruplex was 144 nM. It also bound to the telomere quadruplex, but with lower affinity than to the M1 quadruplex (Kd = 11.5 μM). Lia3 had much higher affinity for either quadruplex probe than for the ss or ds linear forms of the M1 probe (extrapolated Kd over 0.2 mM). In competition experiments, oligonucleotides forming parallel G4 DNA (M1 or M2) were able to compete away the interaction of Lia3 with the M1 quadruplex, whereas linear oligonucleotides did not compete for binding, which further shows that Lia3 binds specifically to parallel G4 DNA *in vitro* (S7 Fig). It is important to note that addition of LiCl to the binding reaction did not inhibit Lia3 binding to the M1 probe when it was pre-assembled into the quadruplex form (S7 Fig–ss competitor).

Together, our genetic and biochemical analyses indicate that Lia3 binds to a parallel G quadruplex that forms near the boundaries of the IESs flanked by A_5_G_5_ sequences to direct accurate excision. We attempted to directly detect these structures in developing macronuclei using available anti-G4 DNA antibodies without success. We could detect putative G4 DNA in the macronuclei of unmated cells and the parental macronuclei of late stage conjugants, possibly due to the very abundant telomeres (S8 Fig). The failure of this approach may indicate that Lia3 binding masks the G4 DNA epitope in developing macronuclei or that the amount of these structures present is below the level needed for detection with these reagents.

**Fig 7:**
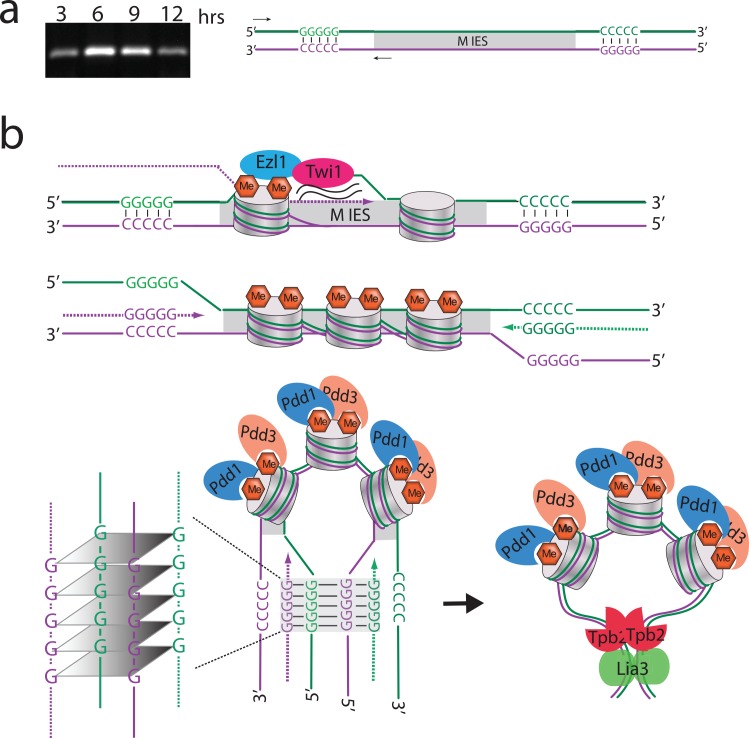
Possible model for how Lia3 determines IES boundaries. (A) RT-PCR across the A_5_G_5_ located at the left boundary of the M IES at 3, 6, 9, or 12 hours post-mixing. (B) Model: Shortly after the new macronuclei form, Twi1 bound to scnRNAs interacts with the nascent transcripts being produced across IES. This interaction recruits the histone methyltransferase Ezl1 to induce H3K9/27me at the IES. After H3K9me3 and H3K27me3 marks have been placed, Pdd1, Pdd3 and other chromodomain containing proteins recognize these marks and induce bending in the DNA causing the two ends of the IES to move towards each other. Around this time, transcription is occurring throughout the developing nuclei including across the polypurine tracts located near the boundaries. This opens up the double helix and allows the single stranded DNA to bind to the newly synthesized messenger RNA. Since the two ends are now in proximity to each other, the RNA/DNA hybrids on each side can bind to each other and form a quadruplex. Lia3 binding to the quadruplex either stabilizes the quadruplex interaction thereby keeping the two ends locked together and facilitating Tpb2 in cutting at the correct location or Lia3 recruits Tpb2 to the correct location through a direct interaction. Dotted lines indicate newly synthesized RNA; solid lines, DNA; hexagons, methylation marks; cylinders, nucleosomes.

Although it is easy to envision how intermolecular association of four oligonucleotides can allow formation of G4 DNA in our gel shift assays, how a four-stranded structure might form at chromosomal loci given that each side of the IES contains a single run of Gs is less obvious. We investigated one possibility, that two of the four strands could be RNA. Hybrid DNA/RNA quadruplexes can form during transcription [40], and transcription of IESs occurs before their excision [35,41]. Transcription would unwind the flanking G tracts, freeing them to interact with other G-rich strands. In this model, the non-coding transcripts created provide the two additional strands needed to complete this structure. To test whether RNA is available to participate in defining M IES boundaries, we used rtPCR to look for transcripts at the time that the Lia3 protein accumulates (S9 Fig) and detected RNAs that span the A_5_G_5_ tract (Fig 7A). We also found that RNA oligonucleotides with the M1 flanking region sequence can form quadruplexes, and that these RNA quadruplexes can compete for Lia3 binding to the M1 G4 DNA probe (S10 Fig). Although these findings support the possibility that non-coding transcripts participate in controlling the boundaries of eliminated sequences, they certainly do not exclude other mechanisms discussed below.

## Discussion

The polypurine tracts flanking the M IES were first shown to control its excision boundaries 25 years ago [16]. Despite the identification of similarly positioned controlling sequences flanking other IESs, how these diverse cis-acting sequences are recognized has remained a mystery. We show here that Lia3 is required to accurately excise the M IES and other IESs possessing flanking polypurine tracks. Lia3 is a novel protein, expressed exclusively during post-zygotic development. In our efforts to characterize its binding to DNA, we discovered that it binds specifically to parallel G4 DNA formed by the M IES A_5_G_5_ sequence. As both this sequence and Lia3 determine IES boundaries, our data strongly support our hypothesis that G4 DNA can form at internal chromosomal loci (not just telomeres) and define specific regulatory domains.

Although we were not surprised that the A_5_G_5_-containing oligonucleotides we used as EMSA probes could form G4 DNA, we did not expect that Lia3 would preferentially bind this structure. Each side of the IES has a single G_5_ tract, and formation of a quadruplex would require four independent copies to come together. The simplest way we can envision this forming *in vivo* involves the transcription from the C_5_ strand, providing G_5_-containing RNA copies that participate in quadruplex formation such that the quadruplex includes the G_5_ DNA tracts on each side of the IES and the two RNA strands (Fig 7B). Four strands of the flanking regulatory A_5_G_5_ DNA are also produced during a round of DNA replication that precedes DNA elimination (Doerder and Debault 1975). If RNAs are not part of the quadruplex, it is likely that the G tracts on each side of the IES from both sister chromatids form the G4 DNA structure. It is also possible that the G tracts of different A5G5-controlled IESs interact to form a single quadruplex.

By showing that Lia3 is both a parallel G4 DNA binding protein and a specific regulator of the excision of IESs containing A_5_G_5_ tracts, we report a compelling case for a role for non-canonical DNA structures in regulating genome organization. The G4 DNA bound by Lia3 appears to bring together G tracts located on each side of the IESs. The formation of G4 DNA through the association of distal G tracts located on different DNA strands is not the obvious outcome, and therefore our findings elucidate an unforeseen potential of dispersed, G-rich DNA sequences to interact. The proposed involvement of transcription in this structure serves two purposes: to unwind the DNA to allow the G tracts to interact with distal partners, and to provide additional G-rich strands to promote a four-stranded structure to form. If such a predicted structure forms *in vivo*, Lia3’s ability to bind these structures may permit this protein to serve as a probe for such structures in genomes beyond *Tetrahymena*. Long non-coding RNAs and non-genic transcription appear to be prevalent in genomes. The model we present in Fig 7 suggests a novel mechanism for these RNAs to interact with DNA and affect chromosomal DNA organization. We believe the ability of Lia3 to bind novel quadruplex structures represents another case in which studies of ciliate genome rearrangements have uncovered new regulatory potential in eukaryotes.

To assist the quadruplex formation between distal G tracts, we propose that the formation of heterochromatin (i.e., establishment of H3K9me3 and H3K27me3) across the IES and subsequent binding of chromodomain-containing proteins Pdd1 and Pdd3, and other DNA excision proteins, position the IES flanking regions in proximity to one other. This organization of IES chromatin aids the formation of the quadruplex, which is stabilized by Lia3 binding. This proposal is consistent with data showing that G4 DNA is enriched in the heterochromatic regions in *Drosophila* polytene chromosomes [28]. The interaction of distal G tracts on different strands represents a novel mechanism to partition chromosomal loci into distinct domains. Once bound, Lia3 guides the domesticated transposase Tpb2 to preferential boundary sites either by directly interacting with the transposase or simply preventing it from cutting elsewhere.

Multiple results from mutational analyses provide evidence that cis-acting sequences on each side of an IES interact with one another. For instance, deletion or other disruption of the boundary-controlling sequence on one side of an IES did not lead simply to inaccurate specification of the boundary on the mutated side of the IES, but instead severely decreased overall rearrangement efficiency [16,17]. Furthermore, chimeric IESs containing one M and one R IES flanking sequence did not exhibit excision at the native M and R boundaries present in the construct, but instead used the native M boundary on one side and a novel boundary that is 45–50 bp away from a cryptic A_5_G_5_ tract present by chance within the R IES sequence [16]. These data are consistent with our model in which G_5_ tracts on each side of the IES come together to form parts of a common structure. Coupled cleavage on both sides of an IES may have been selected for during the domestication of the Tpb2 piggyBac transposase to ensure accurate excision and prevent aberrant double-strand breaks during genome-wide DNA elimination events. Coordinated cleavage on both sides of an IES occurs in the ciliate *Paramecium* [42,43], which also uses a domesticated piggyBac to perform its genome rearrangements [44], indicating that communication between IES ends is a conserved mechanism.

Although we favor a model in which IES heterochromatin is established, and subsequent organization of this chromatin structure helps to bring distal A_5_G_5_ sequences together to form a G quadruplex, we cannot rule out the possibility that Lia3 stabilizes this structure prior to the completion of these chromatin modifications and acts to limit the spread of small-RNA-directed heterochromatin. In the future, we will determine the enrichment of H3K9me3 and H3K27me3 across the developing genome in the presence and absence of Lia3 to assess whether the cis-acting sequences that control IES boundaries actually serve as barrier elements blocking the spreading of chromatin modifications.

Only a subset of IESs have flanking A_5_G_5_ tracts and are controlled by Lia3; yet there are thousands of IESs, which vary greatly in size and sequence, that are faithfully excised during differentiation of the somatic genome. The adjacent M and R IESs are known to use functionally distinct boundary-controlling sequences [16,17]. The use of distinct cis-acting sequences by neighboring IESs would prevent aberrant elimination events between the distal ends of adjacent IESs. Some likely candidates to define the ends of the non-A_5_G_5_ IESs are three *Tetrahymena* proteins with homology to Lia3. Like *LIA3*, each is expressed exclusively during post-zygotic development [45], and the two of which that we have examined localize to developing macronuclei (S11 Fig). Although these Lia3-like (*LTL*) proteins do not have obvious homologs in other organisms, database annotation indicates that their amino termini possess similarity to DNA binding proteins [46]. In our preliminary investigations, disruption of *LTL1* (Ttherm_00499370) results in aberrant excision of several non-Lia3 regulated IESs. It will be interesting to determine whether these related proteins control the boundaries of other IESs by binding to other non-canonical DNA structures. Their study could provide evidence for novel mechanisms used to bring together cis-acting sequences to define specific regulatory domains within genomes.

## Materials and Methods

### Strains and growth conditions

*Tetrahymena* cells were grown at 30°C in either SPP or Neff’s medium under standard conditions [47,48]. Strains CU428 (Mpr1-1/Mpr1-1 [VII, mp-s]), B2086 (II), and CU427 (Chx1-1/Chx1-1 [VI, cy-s]), B*VI (VI), and B*VII (VII), were used to construct knockout strains or were transformed with rDNA constructs. Strains B-VII-427(Chx1-1/Chx1-1 [VII, cy-s]) and B2086 were used for excision assays because both contain only the Δ0.9 form of the M IES in their macronuclei. To promote synchronous mating, cells were starved at 30°C overnight in 10 mM Tris, pH 7.5, prior to mixing at equal cell densities (~2.5x10^5^ cells/ml).

### Generation of transgenic *Tetrahymena*

#### *ΔLIA3* lines

Knockout construct pLIA3KO was generated by amplifying genomic regions upstream of the *LIA3* locus (nucleotides 319498–320510, scaffold scf_8254658) and downstream coding and flanking region (nucleotides 322167–323496, scaffold scf_8254658) with oligonucleotides Lia3_upko5’A plus Lia3_upko3’r and Lia3_dsko5’A plus Lia3_dsko3’r, respectively (S2 Table). Lia3_upko3’r and Lia3_dsko5’A contained complementary linker sequences on their 5’ ends, which created an overlap region between these initial PCR products. These DNA fragments were mixed together with the upstream and downstream most primers and a fused DNA fragment was amplified and cloned into pCR2.1, mediated by Topoisomerase (Invitrogen, Carlsbad, CA). The Neo3 selectable marker from pENTR-D-*MTT1/NEO3* [49] was then inserted as a *BsrG*I-*Asc*I fragment into the complementary linker between the upstream and downstream homology regions. CU428 and B2086 were mated and pLia3KO, linearized using *Acc*65I, was introduced into cells 3hrs into mating by biolistic transformation to obtain micronuclear transformants. Transformants were selected by growth in SPP plus 1 μg/ml CdCl_2_ and 80 μg/ml paromomycin sulfate. Germline transformants were mated with B*VI and B*VII to generate exconjugants with homozygous micronuclei and then crossed to produce complete *ΔLIA3* lines as described for making *ΔDCL1* strains [50].

Southern blot analysis was performed as described [50] and used to confirm replacement of the *LIA3* gene with the Lia3-Neo3 knockout cassette. Genomic DNA was digested with HindIII, fractionated on a 1% agarose gel, transferred to nylon membrane and hybridized with a radiolabeled SpeI-BsrG1 DNA fragment isolated from pLia3KO.

### Survival analysis

Individual pairs, >6 hrs after initiating mating, were transferred to individual drops of SPP and allowed to complete conjugation. Drops containing living cells after 2 days were transferred to 96 well plates containing starved CU428 or B2086. Throughout the day wells were screened for mating pairs. Wells containing paired cells indicated that the initial drop plates had contained back-outs instead of progeny as progeny would not be sexually mature yet. Survival was scored as the percent of drops containing progeny versus the number of drops plated. To score conjugation endpoint, cells were fixed with 2% paraformaldehyde 24 hrs into mating and stained with DAPI.

### RT-PCR

Total RNA (4μg), isolated by RNAsol extraction [51], was converted to cDNA using SuperScript II reverse transcriptase as described [50]. PCR was performed using Lia3rt_FW and Lia3rt_RV primers (S2 Table) to monitor *LIA3* expression and HhpI_FW and HhpI_RV primers (S2 Table) to monitor HhpI expression as a loading control.

### IES excision analyses

gDNA was isolated from mating cells at indicated times. Detection of IES junctions was performed by using PCR primers that amplify across the IES junction as described [2]. Detection of excised IES circles was performed by nested PCR using primers (S2 Table) pointing outward from the IES [52] [53]. PCR products were gel isolated and then TA cloned prior to sequencing.

### Plasmid-based rearrangement assays

Electroporation of wild-type or *ΔLIA3* mating cells with IES-containing rDNA vectors was performed as described [17,54]. Plasmids containing M or R IES sequences or chimeric IESs (pMgtwM_m, pMgtwM_r, pRgtwR_m, pRgtwR_r) were created by first replacing the IES sequence with a gateway recombination cassette then recombining the desired IES sequence into the desired vector. For Southern blot analysis, three individual paromomycin-resistant progeny lines, obtained after electroporation of wild-type or mutant mating cells with IES-containing vectors, were co-cultured for genomic DNA isolation. Ten μg of each DNA preparation was digested with either *Not*I or *Bam*HI, fractionated on 1% agarose gels, transferred to nylon membranes and hybridized to M or R IES (from pDLCM3 or pDLCR5, respectively) [55].

### Purification of Lia3

DNA encoding an N-terminal His tagged Lia3 (His-Lia3) was codon optimized for expression in *E*. *coli* by Life Technologies. His-Lia3 was cloned into *Nco*I and *Xba*I sites of pBAD (a gift from Dr. R. Kranz, Washington University) to make pBAD-HisLia3. His-Lia3 was expressed in *E*. *coli* strain BL21(DE3). After reaching an OD_600_ ~0.8, 0.2% wt/vol Arabinose was added and cells continued to grow for 4hrs before harvesting. Cell pellet was resuspended in native lysis buffer (50 mM NaH_2_PO_4_, 300 mM NaCl, 10 mM Imidazole, and protease inhibitors) and lysed using a French press (1200 psi). Cells were centrifuged at 40,000 rpm for 45min at 4°C and the pellet was resuspended in denaturing buffer (100 mM NaH_2_PO_4_, 10 mM Tris-HCl pH 7.5, 8M Urea, and protease inhibitors) and stirred on ice for 1 hr. Cell lysate was spun at 10,000 x g for 30 min and the supernatant was incubated with Ni-Nta resin for 1 hr before loading onto the column. After washing with 50 mM NaH_2_PO_4_, 300 mM NaCl, and 20 mM Imidazole, proteins were eluted in 50 mM NaH_2_PO_4_, 300 mM NaCl, and 125 mM Imidazole and subsequently dialyzed against 25 mM Tris pH 7.5, 100 mM KCl, 1 mM DTT, 1 mM EDTA, and 10% glycerol before storage at -80°C.

The His-Lia3 coding sequence was PCR amplified using oligonucleotides listed in S2 Table to add an N-terminal TEV protease cleavage sites and *Bam*HI and *Hin*dIII sites. The amplified DNA was cloned into the pMAL-C2X expression vector (New England Biolabs, Ipswich, MA) to create pMAL-TEV-hisLIA3. The plasmid was transformed into BL21(De3) cells and the recombinant protein was purified by using an amylose resin as described [56] and then dialyzed against 25 mM Tris pH 7.5, 100 mM KCl, 1 mM DTT, 1 mM EDTA, and 10% glycerol before storage at -80°C.

### Oligo preparation for gel shifts

Oligonucleotides (Table 1) were labeled by incubation with T4 PNK and [γ -^32^P] ATP for 1 hr at 37°C and then purified using Roche oligo spin columns. Oligos were made double-stranded by mixing equal amounts of complementary oligonucleotides in 10 mM Tris pH 7.5, 5% glycerol, and 100mM LiCl and boiling for 5 min, followed by slowly allowing the oligonucleotides to cool to RT. Prior to gel shifts, oligonucleotides were boiled for 5 min in 10 mM Tris pH7.5, 5% glycerol, and either 100 mM KCl or 100 mM LiCl and slow cooled to RT to allow structures to form.

### Electrophoretic mobility shift assays

For binding and competition experiments, 50–400 nM His-Lia3, MBP-Lia3, or MBP-MS2 was incubated with unlabeled competitor oligonucleotides (Table 1) in binding buffer (10 mM Tris pH 7.5, 1 mM EDTA, 0.1 mM DTT, 5% vol/vol glycerol, 0.010 mg/ml BSA, and either 100 mM KCl or 100 mM LiCl) for 15 min at RT. KCl was used in all binding reactions except when LiCl was use to limit quadruplex formation. Followed by addition of 4 nM ^32^P-labeled oligonucleotide and incubation for another 15 min at RT before 4 μl was loaded onto a 4.5% polyacrylamide (75:1 acrylamide:bisacrylamide) gel. After pre-running gels at 140V for 30 min, samples were fractionated by electrophoresis at 140V for 1 hr 45 min. Gels were subsequently vacuum dried for 1 hr prior to exposure to X-ray film or to a Phosphorimager screen. For binding curve experiments, 4 nM ^32^P-labeled oligo was incubated with 0, 50, 75, 100, 150, 200, 250, 300, 400, 500, 700, 900, 1100, 1300, 1500, 1900, 2100, 2500, or 3000 nM His-Lia3 for 20 min at RT prior to loading on gel.

### Circular dichroism

Oligonucleotides were boiled for 5 min in 10 mM Tris pH 7.5, and either 100 mM KCl or 100 mM LiCl and slow cooled to 4°C. The CD spectra were recorded on a J-810 spectropolarimeter (Jasco). The measurements were carried out with 500 μL 3 μM ODN samples at 4°C under nitrogen. Spectra shown are the average of 3 scans in a range from 220 to 300 nm with a band width of 1 nm, response time of 0.5 s, data pitch of 0.2 nm, and scan speed of 50 nm/min. A blank sample of 10 mM Tris pH 7.5 with 100 mM KCl or 100 mM LiCl was used for baseline correction.

### Fluorescent microscopy

Strains CU428 and B2086 were starved in 10 mM Tris-HCl (PH 7.5) and mixed to induce mating. Between 9 and 10 hours post-mixing, mating *Tetrahymena* cells were fixed in 3% PHEMS-paraformaldehyde essentially as described [57], incubated overnight with a 1:200 dilution of anti-G4 antisera (1H6- EMD Millipore, Billerica, MA) [27], and detected with Alexa 488-conjugated, goat, anti-mouse antisera. Cells were counterstained with DAPI (4',6-diamidino-2-phenylindole) and imaged on a Nikon E600 epiflourescent microscope equipped a Retiga EX CCD camera (Q imaging, Burnaby. B.C. Canada) with Openlab acquisition software v404 (Improvision).

## Supporting Information

S1 Fig Southern blot confirmation of *LIA3* disruption. Representations of wild-type and *ΔLIA3* alleles are illustrated on the right. The *LIA3* gene was replaced with the *NEO3* selectable marker, which confers paromomycin resistance upon induction with CdCl_2_. The HindIII (HIII) restriction enzyme cut sites used to differentiate these two alleles, and the expected sizes of genomic DNA fragments are indicated above each diagram. The region of DNA that was radiolabeled and used as a probe is also shown. The first lane contains genomic DNA from parent strain CU428; the remaining lanes are DNA isolated from: initial germline-transformants after phenotypic assortment (1B2 and 1C2); germline homozygous lines resulting from crossing B*VI to 1C2; complete macronuclear (Mac) and micronuclear (Mic) *ΔLIA3* lines resulting from crossing lines 5A and 5B. An additional high-molecular weight DNA fragment in lines 1A and 1B result from transgene deletion (tgd) occurring in the final cross to make the homozygous ΔLIA3 lines, which removes the HindIII within *NEO3*. The Mic and Mac genotype of each strain is indicated at the bottom of each lane. The migration of Lambda DNA (PstI digest) size markers of specific sizes is given on the left.
(EPS)

S2 Fig Δ*LIA3* lines aberrantly rearrange IES flanked by A_5_G_5_. PCR amplification of IES rearrangement junctions in progeny of wild-type and Δ*LIA3* matings as indicated. (a) IES 54 is flanked by A_5_G_5_ sequences and shows aberrant rearrangement. Lanes 1–4 are from the wt and ΔLIA3 parent lines used for the matings in lanes 5–13. (b-f) PCR across rearrangement junction of IES that rearrange normally in Δ*LIA3* matings. Larger black arrows, pcr primers; asterisks, polypurine tracts; grey boxes, flanking sequences; white boxes, IES; smaller black arrows; predominant wild-type products; grey arrowhead, non-specific PCR products. IES 92 has a sequence resembling a polypurine tract only on its right flank. Although some IES rearrangement is highly variable, those without flanking A_5_G_5_ sequences show similar variability in Δ*LIA3* and wild-type progeny lines.
(EPS)

S3 Fig Isolation of his-tagged Lia3 from *E*. *coli*. (a) Western blot of the dialyzed final isolated protein sample detected using anti-6xhis antisera. Lane 1 is the size marker and lane 2 is the isolated His-Lia3 sample. (b) Coomassie from same gel as (a) confirming that His-Lia3 is the primary protein in the sample. For A and B, arrows point to His-Lia3.
(EPS)

S4 Fig M1 and M2 oligos form G-quadruplexes *in vitro*. (A) M1 and M1-C3 oligos were boiled in 10mM Tris with or without 100mM KCl or 100mM LiCl, slowly cooled to room temperature and then run on a 10% native gel. Black arrows point to the quadruplex form, grey arrows to the ss linear form. (B) CD spectra for M1 and M2 oligos in different salt conditions. M1 and M2 display characteristic spectra for parallel G quadruplex in 100mM KCl but not in 100mM LiCl or when the A_5_G_5_ tract has been mutated.
(EPS)

S5 Fig Isolation of MBP-Lia3. Decreasing amounts (3-fold serial dilution) of MBP-Lia3 (top panel), MBP-MS2 (middle panel), or BSA (bottom panel), were loaded into four wells, fractionated by SDS-PAGE, and visualized by coomassie staining. A known amount (4.0–0.15 ug) of BSA was loaded and used to estimate the amount of purified Lia3 and MS2 protein.
(EPS)

S6 Fig Lia3 binds the M1 boundary sequence when present as G4 DNA. Purified Lia3 protein was incubated with 30 base oligonucleotides corresponding to the M1 boundary (a-c) or the *Tetrahymena* telomere (d) sequence. Increasing concentrations of protein, from 50 to 3000 nM, were added to labeled probes (4 nM). (a) M1 probe DNA in 100 mM KCl allowing quadruplex formation (G4). (b) ssM1 probe DNA or (c) dsM1 annealled with its complementary sequence in 100 mM LiCl preventing quadruplex formation, binding reactions contained 100mM LiCl in place of KCl. (d) *Tetrahymena* telomeric DNA in 100 mM KCL allowing quadruplex formation. The position of migration unbound (ss, ds, or G4 DNA) probe and Lia3 bound probe are indicated by arrowheads.
(EPS)

S7 Fig Only G4 DNA effectively competes for Lia3 binding. Competition gel shift in which different unlabeled competitor oligonucleotides (G4-M1, ssM1, dsM1, mut-M1, G4-M2, or ssM2) are incubated with His-Lia3 at either 5 fold(X) or 20X excess relative to the amount of ^32^P-labeled G4-M1. Black arrows point to the bound M1 probe; grey arrows point to the unbound ss or G4 form of the probe. LiCl was substituted for KCl in binding reaction with non-G4 competitors.
(EPS)

S8 Fig G4 DNA is detectable in macronuclei, but not other nuclei. Immunofluorescent detection of G4 DNA in unmated (A) and 9–10 hour mating (B) *Tetrahymena* cells was performed using a 1:200 dilution of anti-G4 antisera (1H6- EMD Millipore, Billerica, MA) [27] on cells fixed in 3% PHEMS-paraformaldehyde essentially as described [57] and detected with alexa 488 goat, anti-mouse antisera. Cells were counterstained with DAPI (4',6-diamidino-2-phenylindole) and imaged on a Nikon E600 epiflourescent microscope. White arrows point to macronuclei, white arrowheads indicate micronuclei, and red arrows denote developing macronuclei. In unmated cells, the antibody recognizes epitopes in macro- but not micronuclei. In post-zygotic mating cells, G4 DNA is observed in parental macronuclei, primarily after program nuclear destruction initiates and the DAPI-staining intensity is diminishing. At late stages of conjugation, generalized fluorescence becomes more peripheral in the parental macronuclei.
(EPS)

S9 Fig Lia3p is present during IES excision. Western blot using anti-HA. A tagged copy of *LIA3* containing an N-terminal HA epitope was integrated into the endogenous locus. A copy of the *neo3* paromomycin resistance selection cassette was incorporated downstream if the coding region to allow selection of biolistic transformants. Replacement of all copies of the untagged LIA3 with the HA-LIA3 allele in macronuclei (M) or both micro- and macronuclei (C) was confirmed by PCR-based tests. Protein was isolated from either a somatic (M) or complete (C) knock-in of Hemaglutanin (HA)-tagged *LIA3* line matings and fractionated by SDS-PAGE. Arrow points to HA-tagged Lia3. Hours indicate times post-mixing that protein was extracted.
(EPS)

S10 Fig An RNA quadruplex competes for Lia3 binding to G4. Competition gel shift in which different unlabeled RNA corresponding to the M1 flanking DNA was incubated with His-Lia3 at either 1, 5, 10, or 20 fold(X) excess relative to the amount of ^32^P-labeled G4-M1. Black arrows point to the bound M1 probe; grey arrows point to the unbound ss or G4 form of the probe.
(EPS)

S11 Fig Three Lia3-like (LTL) proteins are encoded within the *Tetrahymena* genome. A) Schematic diagram of the Lia3 protein denoting an ~100 amino acid region that shares similarity with three other Tetrahymena-encoded proteins: *LTL1*, Ttherm_00499370; *LTL2*, Ttherm_00600360; *LTL3*, Ttherm_00787380. A multiple sequence alignment of the conserved region was generated with ClustlW2 (http://www.ebi.ac.uk/Tools/msa/clustalw2/). Open arrow, LIA3 coding region, shaded box, conserved region; the amino acids of Lia3 aligned are indicated above the diagram. B) Screenshots of microarray expression data from the Tetrahymena Functional Genomics Database [45] reveals *LIA3* and *LTL* genes are expressed exclusively in post-zygotic development. C) and D) Expression of yellow fluorescent protein-LTL C-terminal fusions show that both LTL1 and 3 are localized to developing macronuclei when DNA rearrangement occurs. The coding region of each was inserted into pIGF-gtw to generate N-terminal fusions with Green Fluorescent Proteins (GFP) and introduced into Tetrahymena cells by conjugative electroporation [54]. GFP localization was visualized in mating cells ~8 hours post-mixing on an Nikon E600 epifluoresence microscope as described [50].
(EPS)

S1 Table M element excision junctions in wild-type and Δ*LIA3* progeny. (DOCX)

S2 Table Additional oligonucleotide primers used in this study. (DOCX)
