# Supplementary material for: A Parallel G Quadruplex-Binding Protein Regulates the Boundaries of DNA Elimination Events of Tetrahymena thermophila
Source: PLoS Genet. 2016 Mar 7;12(3):e1005842. doi: 10.1371/journal.pgen.1005842 (PMC4780704; doi:10.1371/journal.pgen.1005842)
Supplement: S1 Table — (DOCX) [file pgen.1005842.s012.docx]

**Supporting Information: S1 Table**

**S1 Table: M element excision junctions in wild-type and Δ*LIA3* progeny**

**M IES excision junctions in wild-type progeny^1,2^**

TTTGATGGta **Δ** attggttaaa 6/8/12 WT1-1

tCcattcaaa **Δ** tTGGTTAAAT 6/8/12 WT1-3

**M IES excision junctions Δ*LIA3* progeny^1.2^**

ttgatggTAA **Δ** TAATTggtta 7/6/11 #1

TTTTGTTCAT **Δ** TTATTGAAAT 7/6/11#3

aaggtacgaT **Δ** AAAGACCCAA 7/6/11#4

AAATTGCTAA **Δ** ATGAATAATT 7/6/11#5

Ttgatggtaa **Δ** ATAATTGGTT 10/31/11 #8-1

GAATAAATTA **Δ** attgaaattc 10/31/11 #13-1

ATTTAAAAAA **Δ** ttgaaattcg 10/31/11 #14-2

AAACATTCaA **Δ** tgaaattcgg 10/31/11 #14-5

atatttaatt **Δ** AAATAAAAAC 10/31/11 #16-1

aacattcaag **Δ** TTTGAAAGTA 6/8/12 KO 5-2

atagatagat **Δ** TTAATTTTTT 6/8/12 KO 5-3

ttatttacac **Δ** TTAAATTGAT 6/8/12 KO 13-1

GAATAAATTA **Δ** attgaaattc 6/8/12 KO 13-4

TTATTTACAC **Δ** ttaaattgat 6/8/12 KO 13-5

**M IES circle junctions in wild-type progeny^1,3^**

tcttcctatt x aaTTTTTatc 1/27/12 WTU1

tgaatgaaTA x ATTAGTATgg 2/01/12 WTU2

CATTAATCAC x aattttgttc 3/02/12 WT14

**M IES circle junctions in *ΔLIA3* progeny^1,3^**

tcttaattaa x TATTTAATTG 1/27/12 KO18-1

taaattaata x TCATTCTTTA 1/27/12 KO24-1

tgaatgaata x atATTTaATT 1/27/12 KO24-3

ttgtatttca x TTGATGCCTT 2/01/12 KO16-1

tttaaacatt x aaTTTATTAA 2/01/12 KO18-2

ttttgcTtac x tttgttCATT 3/02/12 KO16

TATTTGCAtt x aagctaattt 3/02/12 KO18

taaagacata x ATTTATTTAC 3/02/12 KO24

^1^Red bases indicate sequences that are repeated on both sides of the excision junction which makes assignment of those sequences to the right or left side of the IES ambiguous.

^2^The **Δ** symbol represents the M IES region excised between the left and right junction sequences given.

^3^The X denoted the position of the circle join; the sequence given reveals the boundaries of the DNA eliminated from the macronucleus.
