# Supplementary material for: A Parallel G Quadruplex-Binding Protein Regulates the Boundaries of DNA Elimination Events of Tetrahymena thermophila
Source: PLoS Genet. 2016 Mar 7;12(3):e1005842. doi: 10.1371/journal.pgen.1005842 (PMC4780704; doi:10.1371/journal.pgen.1005842)
Supplement: S2 Table — (DOCX) [file pgen.1005842.s013.docx]

**Supporting Information: S2 Table**

**S2 Table: Additional oligonucleotide primers used in this study**

**pLia3KO generation**

Lia3_upko5’A – 5’- AGGTACCTCAGTTAGCTAAAGACCGTCAAA – 3’

Lia3_upko3’r - ggcgcgcctaggtgtacagtcgaCAAACGAAGATACTCGCTGCT

Lia3_dsko5’A – gtcgactgtacacctaggcgcgcCGAGTAGTATACCTCTGAACAGTTT

Lia3_dsko3’r – AGGTACCAGCAATTTCCTCAATCCTACC

**LIA3 Expression analysis**

LIA3 rt-pcr

Lia3rt_FW - ACTCACAATTCGAAGAAGTCAAAGG

Lia3rt_RT – TCAAACTAGCTTGCTGAAATCAT

HHPI rt-pcr

HhpI_rt FW - GGAGCTTCAACTCATTAAACACG

HhpI_rt RV - TCGGGAGAAGCATACTTAGCA

**IES excision analysis**

M IES Junction PCR

M1194 - GTGGGGAGGGAGAAGGATTCAAC

M002 - AGCTTAAACAAATGCCATATTGAG

R IES Junction PCR

R5’(168-sense)-2 - AATTATTCTTTATAATCTGACTC

R3’(1453-anti)-2 - AAGATAGTTCTAGAATAAGAC

IES 97 Junction PCR

97416498_FW - AAAGTTTTCAAAGATTTGTTATCC

97416498_RV – TGTTCTGCTTTAAAATTTTGCTC

IES 57 Junction PCR

57860466_FW - ACAAACAAATAAAGTAGTCCTTTTGA

57860466_RV – AGCTTCTTTGATTGATTTTTGA

IES 55 Junction PCR

55122451_FW - AGCTTTTAAGAAATAGAGAATGAATAA

55122451_RV – TCCATCGCTTTCATAACTTT

TLR IES Junction PCR

Tlr1-FW2.2 - GCTTTACATATAATTATCTGCTTC

Tlr1-RV2 - CGAGTTAAATAAAACATAACTTAGTTAGC

Tlr1-Mic2 - cttaagaaagtgatgaatagatagctg

IES 1 Junction PCR

Win1_3369L-141 - CCAACAAAATGCTAATTAAAATGG

Win1_3369R-62r - TCATATCCTTAATTTGCCTCATA

IES 2 Junction PCR

win1_8019L-126 - CAGGCTGCTAGCTTAAATGGA

win1_8019R-77r – TCACACATTACGGTTTTCTTGC

IES B Junction PCR

IES7_MDSL-112 - GGATTGATTGGCATAAATGGA

IES7_MSDR-158 - AAGCCCAGAATACCGCAGTTC

IES C Junction PCR

IES1_MDSL-110 - TGGAAGATCTACTTCAAAGCGAAT

IES1_MDSR-31 – CCAGCTAGACACCCTGTATCAA

IES 54 Junction PCR

5402864_FW - AAGTTCCTTGAAATATAATTCTTAAACA

5402864_RV – AACATTAATTAAGACCCCCTTTTT

IES 92 Junction PCR

92878309_FW - CACAAGCAACTTGAAAGCAA

92878309_RV - TTTTGAAACACTTTGACTACGC

IES 93 Junction PCR

93642689_FW - TTGAAGCAGTCATAATTTCAA

93642689_RV – TTTTTAATTTTCAAATCTGG

IES 95 Junction PCR

95493965_FW - AAGCTTAAAACAGCTTTATTTTTG

95493965_RV - ACACCAATTTATGAAATGCTAGA

**IES Circle Junction Amplification**

M Circle PCR Round I

M_circle1_RV - CCTTATTAAGTGATCTAAAGACCCAAG

M_circle0.9_FW1 - GAAACCCATCCCCCTTTTT

M Circle PCR Round II

M1001 revised - AAC TTA TTG AAA TTC GGC TAA CATTATG

M_circle0.9_FW2 - TTGTCTTGAATGTTTACAAAAATGTG

R Circle PCR Round I

R_circle1_FW2 - TTTTTCTTGTCTTACTTCAAAC

R round 1 RV - TGAGTATCAAATCTTATTTTAATTG

R Circle PCR Round II

R round 2 FW (RI5-519RC) - TTTAATTAGTCAGGTTATAGG

R round 2 RV (RI3-1317) – CTTAATTCACGTAATCAAGGAC

**Lia3-pMAL expression vector cloning**

TEVHISLIA3_up -CACCGGATCCGAGAATTTGTATTTTCAGGGTACCATGGTACATCATCATCACCATCA

LIA3s_dsHIII -GATATCAAGCTTAGAACAGACTATTCAGCGGAATGCT
